# Effects of oestrogen deficiency on the alveolar bone of rats with experimental periodontitis

**DOI:** 10.3892/mmr.2015.3875

**Published:** 2015-06-02

**Authors:** XIN-CHEN XU, HUI CHEN, XI ZHANG, ZAN-JING ZHAI, XU-QIANG LIU, XIN-YI ZHENG, JUN ZHANG, AN QIN, ER-YI LU

**Affiliations:** 1 Department of Prosthodontics, Shanghai Key Laboratory of Stomatology, Shanghai Jiao Tong University School of Medicine, Shanghai 200011, P.R. China; 2 Department of Orthopaedics, Shanghai Key Laboratory of Orthopaedic Implants, Shanghai Ninth People's Hospital, Shanghai Jiao Tong University School of Medicine, Shanghai 200011, P.R. China

**Keywords:** osteoporosis, periodontitis, ovariectomy, alveolar bone

## Abstract

Periodontitis is an inflammatory disease characterized by loss of connective tissue and alveolar bone, and osteoporosis is a common disease characterized by a systemic impairment of bone mass and microarchitecture. To date, the association between periodontitis and osteoporosis has remained to be fully elucidated. In the present study, an experimental rat model of periodontitis was used to explore the effects of oestrogen deficiency-induced osteoporosis on the maxillary alveolar bone. Forty-four female, six-month-old Sprague-Dawley rats were randomly divided into four groups: Control, ligature, ovariectomized (OVX), and OVX + ligature. One month after ovariectomy, rats in the ligature and OVX + ligature groups received ligatures on their first and second maxillary molars for 1 month. Fluorescent labelling was performed prior to sacrificing the animals. At the end of the experiment, the maxillae and serum were collected and subjected to micro-computed tomography analysis, confocal laser-scanning microscopic observation, Van Gieson's fuchsin staining, tartrate-resistant acid phosphatase staining and ELISA. Ligatures slightly reduced the alveolar bone mineral density (BMD) and bone formation rate, but significantly reduced alveolar crest height (ACH). Ovariectomy reduced the alveolar BMD, impaired the trabecular structure, reduced the bone formation rate and increased the serum levels of bone resorption markers. Animals in the OVX + ligature group exhibited a lower alveolar BMD, a poorer trabecular structure, a reduced ACH, a lower bone formation rate and higher serum levels of bone resorption markers compared with those in the control group. The results of the present study showed that ovariectomy enhanced alveolar bone loss and reduced the ACH of rats with experimental periodontitis. Thus, post-menopausal osteoporosis may influence the progression of periodontitis.

## Introduction

Periodontitis is defined as a bacteria-induced disease that gradually destroys periodontal tissues, including the gums, cementum, periodontal ligaments and supporting alveolar bone (1,2). Data from 2009–2010 showed that almost half of the US population over 30 years of age (47.2%) suffered from a certain degree of periodontitis, including 8.7% with mild disease, 30.0% with moderate disease and 8.5% with severe periodontitis (3). Due to the high prevalence of periodontitis and accompanying loss of teeth or the edentulous jaw, most elderly people regard oral health as an important aspect of life quality for various physical, social and psychological reasons (4).

Osteoporosis is a common disease characterized by systemic bone loss and impaired bone microarchitecture. Post-menopausal women are usually more vulnerable to osteoporosis due to their decreased oestrogen levels that affect bone metabolism (5,6). Guiglia *et al* (7) described a possible association between osteoporosis and periodontal disease, suggesting that osteoporosis may facilitate the alveolar bone resorption caused by periodontitis (7). Specifically, osteoporosis results in an increase in certain inflammatory factors, a number of which also participate in the progression of periodontitis.

In recent decades, numerous studies have focused on the association between osteoporosis and periodontitis at the bone level. Several studies have reported that osteoporosis promotes the loss of periodontal attachment, loss of alveolar bone height and even tooth loss (8–10). Tezal *et al* (11) reported that low skeletal bone mineral density (BMD) is associated with loss of proximal alveolar bone and clinical attachment. By contrast, several studies have suggested that this association was weak (12), and Brennan-Calanan *et al* (13) reported that systemic bone density and oral infection independently influence oral bone loss in post-menopausal women. However, previous studies have also shown a great diversity in sample sizes and measurement methodologies, and most have been retrospective clinical studies (14–17). Thus, whether oestrogen deficiency-induced systemic bone loss jeopardizes alveolar bone remains controversial.

A small number of well-controlled experimental animal studies have investigated the association between osteoporosis and periodontitis. Through histometric analyses and assessment of serum alkaline phosphatase and calcium, Duarte *et al* (18) demonstrated that oestrogen-deficiency may significantly increase bone loss resulting from ligature-induced periodontitis in rats, and that there was a synergistic effect between oestrogen deficiency and plaque accumulation. Amadei *et al* (19) observed a significant increase in bone loss, morphometrically evaluated using photo documentation, when ligation occurred 90 days after rats underwent ovariectomy, suggesting that long-term oestrogen deficiency affects ligature-induced alveolar bone loss. However, other studies were unable to correlate the absence of ovarian hormones with periodontal alterations in rats using radiographic analyses with digital dental X-ray equipment (20). In the present study, using micro-computed tomography (micro-CT) analysis, the effect of oestrogen deficiency-induced osteoporosis on the alveolar bone of rats with experimental periodontitis (EP) was explored. It was hypothesized that oestrogen deficiency-induced osteoporosis not only facilitates alveolar bone loss, but also jeopardizes bone microarchitecture in local alveolar bone.

## Materials and methods

### Animals, treatments and experimental design

Forty-four female, six-month-old Sprague-Dawley rats with a mean weight of 400±30 g were obtained from the Department of Laboratory Animal Science (Ninth People's Hospital, Shanghai Jiao Tong University School of Medicine, Shanghai, China). The rats were housed in a 21°C room with a 12-h light/dark cycle. The general condition of the animals was monitored daily and body weight was recorded weekly. The Ethics Committee and the Animal Care and Use Committee of Shanghai Jiao Tong University School of Medicine (Shanghai, China) approved the experimental protocol and the procedures performed.

After two weeks of adaptation, the rats were randomly divided into four groups, with 11 rats in each group: The control, ligature, OVX and OVX + ligature groups. Rats in the OVX and OVX + ligature groups underwent bilateral ovariectomy, whereas the other animals underwent a sham surgery (21,22). Four weeks later, EP was induced by placing 3/0 silk sutures (Johnson & Johnson Medical, Shanghai, China) subgingivally around the bilateral first and second maxillary molars (M1 and M2, respectively) for four weeks. Ligature placement initiated local inflammation and alveolar crest bone resorption (23,24). To ensure the establishment of EP, the ligatures were checked twice weekly and replaced when necessary. All treatments were conducted under general anaesthesia (10% chloral hydrate; Sigma-Aldrich, St. Louis, MO, USA; 4 ml/kg via intraperitoneal injection).

Each rat was injected intraperitoneally with tetracycline (TE; 25 mg/g), alizarin red (AL; 20 mg/kg), and calcein (CA, 10 mg/kg) (all from Sigma-Aldrich) at 25, 15, and 5 days, respectively, prior to sacrification. At the designated end-point, blood samples were collected by cardiac puncture under anaesthesia and centrifuged (6,500–7,000 × g, 15–20 min) to recover the serum, which was stored at −80°C until analysis. Subsequently, the rats were sacrificed with an overdose of anaesthesia. Bilateral maxillary bone specimens were harvested, fixed in 4% paraformaldehyde (Sigma-Aldrich) for 48 h and transferred to 70% ethanol prior to further testing.

### Micro-CT scanning and assessment of the alveolar crest height (ACH)

**Figure 1:**
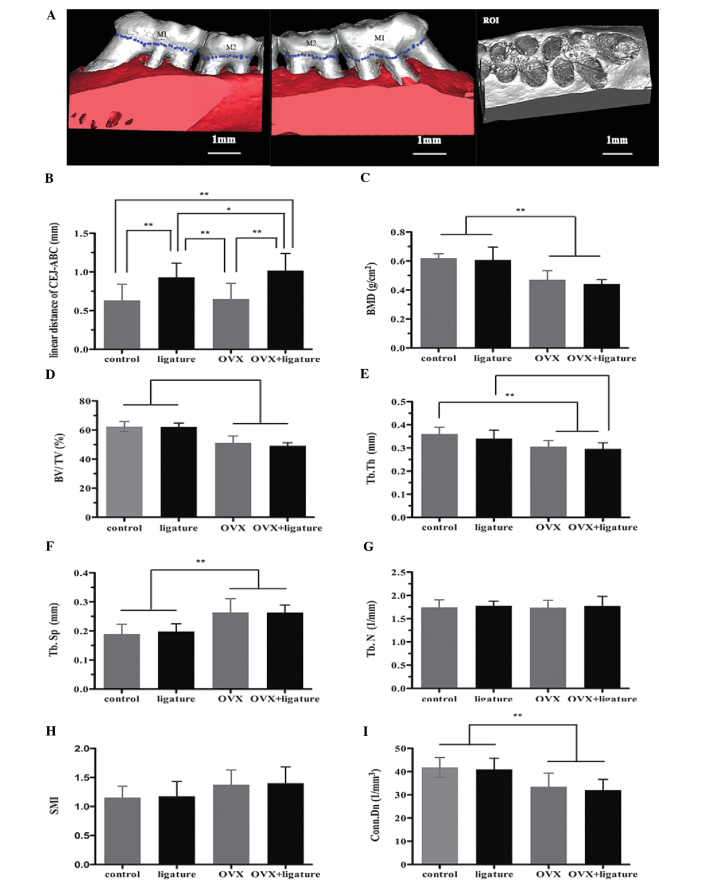
Effects of ovariectomy on ACH and alveolar bone microarchitecture of rats with experimental periodontitis by micro-computed tomography analysis. (A) Buccal and palatal sides of the maxillary alveolar bone, where the ACH was measured between the CEJ and the ABC in the mesiolingual, mesiobuccal, distolingual and distobuccal regions of the M1 and M2. The ROI is a cuboidal bone body that encompasses the M1 and M2 roots. (B) Analysis of the CEJ-ABC linear distance after 1 month of ligation. (C-I) Analysis of micro-computed tomography volumetric parameters: (C) BMD, (D) BV/TV ratio, (E) Tb.Th, (F) Tb.Sp, (G) Tb.N, (H) SMI and (I) Conn.Dn. Values are expressed as the mean ± standard deviation (^*^P<0.05; ^**^P<0.01). OVX, ovariectomy; ACH, alveolar crest height; CEJ, cement-enamel junction; ABC, alveolar bone crest; ROI, region of interest; M1, first maxillary molar; M2, second maxillary molar; BMD, bone mineral density; BV/TV, bone volume/tissue volume; Tb.Th, trabecular thickness; Tb.Sp, trabecular separation; Tb.N, trabecular number; SMI, structure-model index; Conn.Dn, connectivity density.

A cone-beam micro-CT system (Skyscan1176; Skyscan, Kontich, Belgium) at Soochow University Orthopaedic Institute (Suzhou, China) was used to scan maxillary bone specimens. The X-ray generator was set at a voltage of 50 KV, a current of 500 *µ*A and a fixed shutter speed of 900 msec. The images were re-constructed using NRecon (version 1.5.1.4; Skyscan, Kontich, Belgium). Fig. 1A illustrates the region of interest (ROI) for analysis of tooth-supporting alveolar bone in the maxillae. On the basis of a selected Hounsfield Unit (HU) grayscale threshold value, microstructural indicators of BMD, bone volume/tissue volume ratio (BV/TV), trabecular thickness (Tb.Th), trabecular separation (Tb.Sp), trabecular number (Tb.N), structure-model index (SMI) and connectivity density (Conn.Dn) were calculated using CTAn (version 1.10; Skyscan). These measures were used to quantify the bone (BV/TV), determine the average width of the bone structure (Tb.Th), determine the number of traversals across the bone trabeculae per unit length (Tb.N), determine the distance between trabeculae that corresponds to the bone marrow measurement (Tb.Sp), determine the number of trabecular elements that can be removed without changing the bone network and provide an estimate for the number of trabecular connections per mm^3^ (Conn.Dn), and indicate the relative prevalence of rod-like or plate-like trabecular bone (SMI). SMI was defined as an interval between 0 and 3, where 0 is an ideal plate-like structure and 3 is a cylinder.

According to a previous study (25), the linear distance between the cement-enamel junction (CEJ) and alveolar bone crest (ABC) was measured to reflect the decreased volume of alveolar crest height (ACH). The decreased ACH was obtained by averaging the CEJ-ABC distances measured at the mesio-lingual, mesiobuccal, distolingual and distobuccal parts of the specimen. Clinically, a decreased ACH illustrates the integrity of tooth-supporting alveolar bone, with lower CEJ-ABC values reflecting better-quality alveolar bone (26).

### Bone histomorphometry

The left maxillae were sequentially de-hydrated, de-calcified, embedded in methyl methacrylate (Sigma-Aldrich). Sections were prepared from the occlusal surface of the tooth crown to the alveolar bone mesiodistally along the plane parallel to the long axis of the tooth and then cut to 100–200 *µ*m using the Leica SP1600 Microtome (Leica Microsystems, Heidelberg, Germany) and polished to a final thickness of approximately 20 *µ*m by sequential usage of P300, P800 and P1200 sandpaper. To obtain the mineral apposition rate (MAR), fluorescent labeling, which was performed in the live rats as described above, was visualized. A confocal laser-scanning microscope (TCS Sp2 AOBS; Leica Microsystems, Wetzlar, Germany) was used to capture images of the fluorescent labeling line (27). The excitation/emission wavelengths for each of the fluorochromes were 405/580 nm (TE; yellow), 543/617 nm (AL; red) and 488/517 nm (CA; green). Sections were also stained with Van Gieson's fuchsin (Sigma-Aldrich) for histological observation. The tabecular bone dynamic parameters were measured from a 1 mm^2^ box positioned at the interradicular region of M1 (magnification, ×100), using tetracycline and calcein labels. MAR was measured using a Bioquant image analysis system (Bioquant OSTEO II Version 8.12.20, Bioquant Image Analysis Corporation, Nashville, TN, USA).

The right maxillae were de-calcified in 10% EDTA (Sigma-Aldrich) for 2 months and then embedded in paraffin. Serial sagittal sections (5 *µ*m) were stained with osteoclast-specific, tartrate-resistant acid phosphatase (TRAP; Sigma-Aldrich), observed microscopically (Eclipse 90i; Nikon, Tokyo, Japan) and micrographed. ImageJ 1.46r software (National Institutes of Health, Bethesda, MD, USA) was used to analyze the TRAP- and Van Gieson's fuchsin-stained sections. The number of TRAP-positive, multinucleated osteoclasts was counted in each sample.

### Serum bone resorption biomarkers

The serum bone-specific resorption markers, tartrate-resistant acid phosphatase 5 b (TRACP5b) and C-terminal telopeptide of type I collagen (CTX-1), were quantified in all serum samples using rat ELISA kits (SB-TR102 and AC-06F1; IDS, Fountain Hills, AZ, USA).

### Statistical analysis

All values are expressed as the mean ± standard deviation. One-way analysis of variance with Bonferroni's post hoc test was performed to compare the between-group means for all outcomes. The Statistical Package for the Social Sciences, version 17.0 (SPSS, Inc., Chicago, IL, USA) was used for all analyses. P<0.05 or P<0.01 was considered to indicate a statistically significant difference between groups.

## Results

### Effect of ovariectomy on ACH

**Figure 2:**
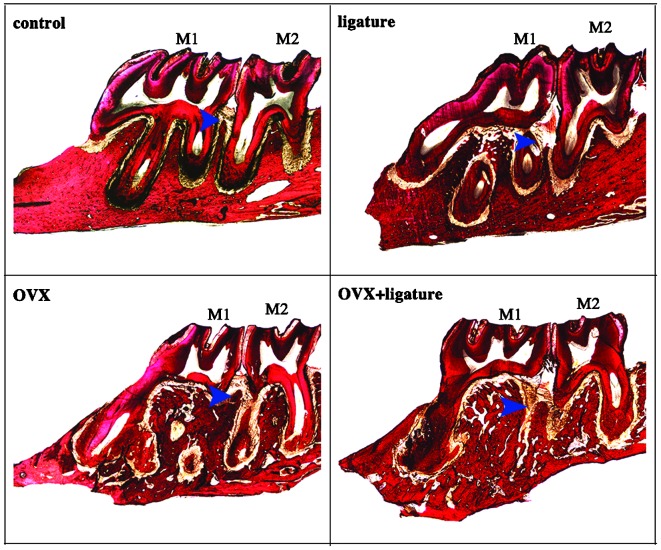
Effect of OVX on alveolar bone in rats with experimental periodontitis, based on a histomorphometric analysis. Images of the maxillary bone surrounding the M1 and M2 after staining with Van Gieson's fuchsin (magnification, ×40). Blue arrows show the alveolar crest in these longitudinal sections. OVX, ovariectomy; M1, first maxillary molar; M2, second maxillary molar.

To obtain ACH values, the linear CEJ-ABC distances at four sites for each specimen were measured and averaged (Fig. 1). The ACH decreased by an average of 0.2979 mm in the ligature group and by 0.3858 mm in the OVX + ligature group, compared with that in the control animals (both P<0.001; Fig. 1B). Compared to that in the OVX group, the ACH decreased by 0.2814 mm in the ligature group and by 0.3693 mm in the OVX + ligature group (P<0.001; Fig. 1B). Statistical analysis of the comparison between the control and OVX groups indicated that ovariectomy did not have any effect on the ACH of non-ligature rats. However, ovariectomy promoted alveolar bone resorption, affecting the ACH in the rats in the OVX + ligature group, as indicated by the lower ACH in the OVX + ligature group compared with that in the ligature group (P=0.042; Fig. 1B). Furthermore, an obvious ACH decrease in the ligature group and in the OVX + ligature group was observed between M1 and M2 in the Van Gieson's fuchsin-stained sections (Fig. 2; blue arrow).

### Effect of ovariectomy on alveolar bone microarchitecture

Based on the analysis of the ROI in the alveolar bone, OVX rats exhibited lower BMDs than non-OVX rats (P<0.001; Fig. 1C). A decreased BV/TV ratio was associated with a reduced Tb.Th as well as an increased Tb.Sp in the OVX and OVX + ligature groups compared with those in the control and ligature groups (P<0.001; Fig. 1D-F). There was also a significant decrease in the Conn.Dn in the OVX and OVX + ligature groups, whereas the Tb.N and SMI remained relatively consistent among the groups (Fig. 1G-I). Fig. 2 shows the bone profile in a longitudinal section. Obvious osteolysis was detected in the OVX rats, which was not present in the ligature-only group.

### Effect of ovariectomy on alveolar bone MAR and the number of active osteoclasts

**Figure 3:**
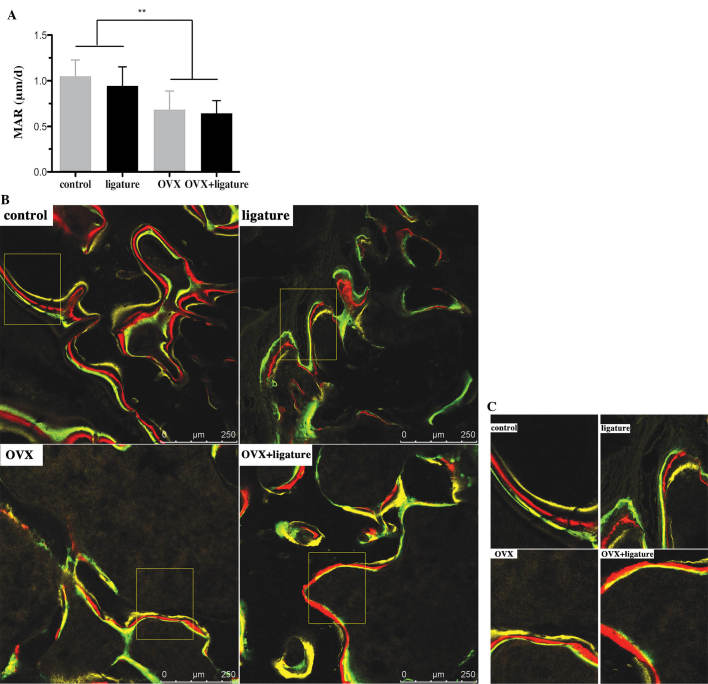
Effect of OVX on the MAR in rats with experimental periodontitis, based on fluorescent labelling. (A) Analysis of MAR. Images of fluorescent labeling was captured using a confocal laser-scanning microscope and MAR was measured using a Bioquant image analysis system with a 1 mm^2^ box positioned in the inter-radicular region of M1 at a magnification of ×100, using tetracycline and calcein labels (^**^P<0.01). (B) Images of sequential fluorescent labelling of the alveolar bone, showing the results of tetracycline (yellow), alizarin red (red) and calcein (green) staining (magnification, ×100). (C) Magnified excerpts of B indicated by boxes (magnification, ×200). The space between two labels represents newly mineralized bone that developed during the 10-day period between label injections. MAR, bone mineral apposition rate; OVX, ovariectomy.

Due to the oestrogen deficiency resulting from ovariectomy, significantly lower MAR values were observed in the OVX rats compared with those in the non-OVX rats (P<0.001; Fig. 3A). EP mildly aggravated the trend, as indicated by the slightly lower MAR value in the OVX + ligature animals than that in the OVX-only group. The yellow, red and green fluorescent labelling lines were intertwined in the OVX + ligature group, in contrast to the obvious separation between the lines in the control group (Fig. 3B and C).

**Figure 4:**
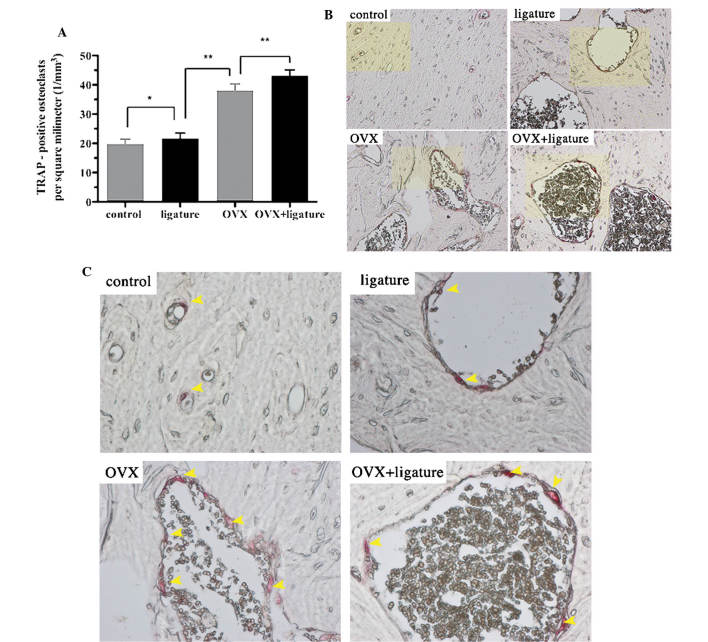
Effect of OVX on osteoclast numbers and activity in rats with experimental periodontitis, based on TRAP staining. (A) Analysis of the number of osteoclasts in a 1-mm^2^ area. Values are expressed as the mean ± standard deviation (^*^P<0.05; ^**^P<0.01) (B) Images of alveolar bone after TRAP staining (magnification, ×200). (C) Magnified areas from B highlighted in a yellow (magnification, ×400). Multinuclear osteoclasts are stained red (yellow arrows). TRAP, tartrate-resistant acid phosphatase; OVX, ovariectomy.

Analysis of the TRAP-stained sections showed that the number of osteoclasts was increased in the ligature, OVX and OVX + ligature groups by 9.59, 92.42 and 117.42%, respectively, compared to that in the control group (Fig. 4A). In addition, variations in the shape and location of the osteoclasts were also found among the four groups. In contrast to the control and ligature groups, the OVX group and particularly the OVX + ligature group exhibited larger, irregularly shaped osteoclasts and more eroded bone surfaces (Fig. 4B and C; arrows).

### Effect of ovariectomy on serum levels of TRACP5b and CTX-1

**Figure 5:**
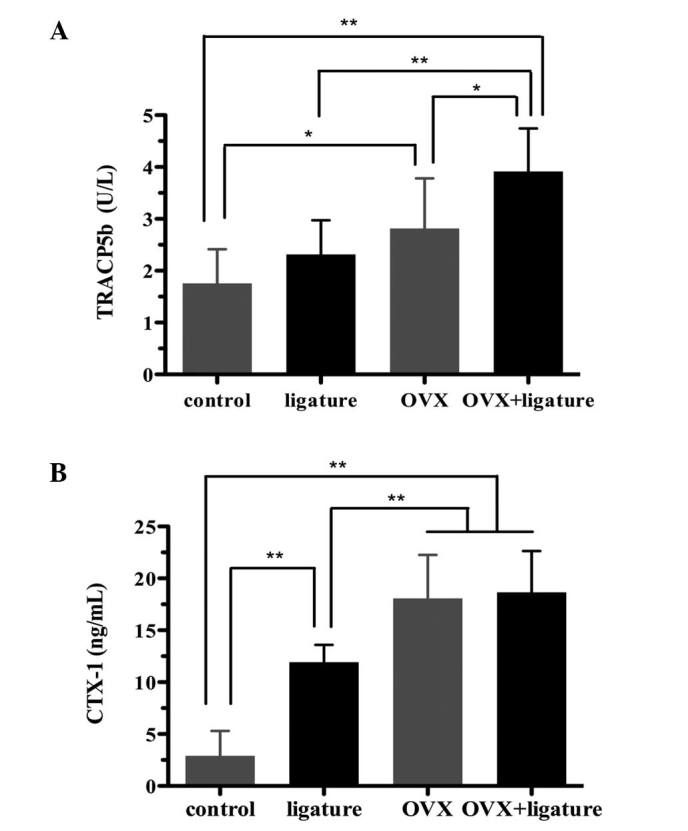
Effect of OVX on the serum biochemical markers (A) TRACP5b and (B) CTX-1 in rats with experimental periodontitis. TRACP5b and CTX-1 were quantified using ELISA and the values are expressed as the mean ± standard deviation (^*^P<0.05; ^**^P<0.01). TRACP5b, tartrate-resistant acid phosphatase 5 b; CTX-1, C-terminal telopeptide of type I collagen; OVX, ovariectomy.

The OVX + ligature group showed elevated serum TRACP5b levels as compared with those in the OVX (P=0.017), ligature (P<0.001) and control (P<0.001) groups, as well as elevated serum CTX-1 levels compared to those in the ligature (P=0.001) and control (P<0.001) groups. In addition, the OVX group showed increased serum TRACP5b levels compared to those in the ligature (P=0.921) and control (P=0.030) groups, as well as increased serum CTX-1 levels compared with those in the ligature (P=0.001) and control (P<0.001) groups. Furthermore, the ligature group showed significantly higher serum CTX-1 levels (P<0.001) and slightly higher TRACP5b levels (P=0.839) compared with those in the control group (Fig. 5).

## Discussion

Extensive epidemiologic and experimental studies have proven the existence of systemic risk factors pertaining to the initiation, progression and severity of periodontitis (28,29). A novel concept also recognizes that systemic risk factors may determine the rate of progression, age at onset and severity of periodontal disease (30). Among these risk factors, osteoporosis is one of the six main factors (30). In addition, periodontitis and osteoporosis are major health problems among the elderly (31). Therefore, an explicit understanding of the correlation between the two diseases is critical to the prevention of the morbidity and mortality associated with the disorders, particularly in the elderly. However, additional clarification regarding the association between osteoporosis and periodontitis is still required, in addition to an understanding of the extent to which osteoporosis contributes to the overall risk of periodontitis. To better understand these associations, experimental animal models are valuable for initial investigations. Sex steroid deficiency, particularly that of oestrogen, is considered to be the main cause of osteoporosis, and OVX rats are widely used as animal models of osteoporosis (18,20,32). In addition, the periodontal tissue of rats resembles that of humans (33).

Alveolar bone is an important component of dental treatments, including prosthodontic, implant and orthodontic treatments (31,34). When analyzing bone mass and volumetric bone parameters, the present study identified that ovariectomy had a significant influence on all parameters except Tb.N and SMI, whereas local simulation of periodontitis failed to significantly change these parameters. This observation was in accordance with a previous study (19), which showed that ligation at 90 days after ovariectomy induced a significant increase in bone loss compared with that in the ligature only group. However, ligatures concurrent with ovariectomy only slightly aggravated the alveolar bone loss, compared with that following ovariectomy alone. The ACH is a measure for the evaluation of periodontitis (9,13). In the present study, ovariectomy alone did not reduce the ACH, but aggravated the reduction in ACH in combination with ligatures. Low alveolar BMD and impaired alveolar bone structure may lead to increased bone resorption of the alveolar crest when periodontitis occurs concurrently with ovariectomy. In addition, ovariectomy-induced oestrogen deficiency disturbs the cytokines that may increase host susceptibility to infection, which also facilitates the progression of periodontitis (31,35–37).

The coupling of bone resorption and bone formation is of great significance to bone metabolism, which is affected by hormones and cytokines (38). In the present study, the bone formation rate was lower than the bone resorption rate in OVX rats, in contrast to the balanced parameters in non-OVX rats, which could be observed from the MAR rate. In contrast to the control and ligature groups, the MAR rate decreased in the OVX and OVX + ligature groups, while there was no statistical difference between the control group and ligature group. The mixed fluorescent labelling line and the reduced MAR of the OVX rats indicated that oestrogen deficiency-induced osteoporosis influenced the balance of alveolar bone formation and resorption. However, no significant MAR reduction was observed in the ligature rats, which may be due the time required for bacterial accumulation prior to impacting bone metabolism after ligation. The local accumulation of bacteria and bacteria-derived factors may stimulate a local inflammatory reaction and activation of the innate immune system. The immune cells secrete cytokines that promote osteoclast maturation, leading to an imbalance in bone metabolism (1). However, these processes take time to occur. Therefore, rats in the OVX + ligature group showed the lowest MAR among the four groups due to the synergic effect. In the present study, the number of TRAP-positive osteoclasts demonstrated an increasing trend in the ligature, OVX and OVX + ligature groups. However, ligature-induced periodontitis led to a significantly increased number of osteoclasts. Furthermore, the levels of two specific serum bone resorption markers, TRACP5b and CTX-1, increased in the ligature rats, but not in the OVX rats. Interleukin (IL)-6 and IL-1 in bone marrow cells are known to stimulate osteoclastic bone resorption under conditions of oestrogen deficiency (39).

Periodontitis is a disease normally caused by bacterial infection, which produces factors or antigens that stimulate local inflammatory reactions and activities of the innate immune system (1). Previous studies have suggested that cyto-kines, including IL-17 or tumour necrosis factor-α (TNF-α), have a key role in periodontal alveolar bone resorption (40–43). IL-17 exerts its osteoclastogenic activity by enhancing receptor activator of nuclear factor kappa-B ligand expression in osteo-blasts and CD4^+^ T cells, and promotes alveolar resorption when released in excessive amounts (44–46). TNF-α directly contributes to periodontal damage through its effect on osteoclastogenesis, through amplification of inflammatory immune reactions and through the inhibition of differentiation and bone nodule formation (47–49). Thus, oestrogen deficiency, associated with increased systemic levels of IL-17 or TNF-α, may augment the local levels of these factors in the alveolar bone and facilitate alveolar bone resorption. This concept is in accord with previously presented results that suggested low oestrogen induced T- and B-cell abnormalities, increased local production of the bone-active cytokines, and finally resulted in periodontitis progression (50).

In conclusion, the present study demonstrated that ovariectomy resulted in the deterioration of the alveolar bone microarchitecture, ACH reduction, decline in the bone formation rate and increased osteoclast activity. These observations indicated that post-menopausal osteoporosis impacts the progression of periodontitis.
